# Shortened time to diagnosis for patients suspected of urinary bladder cancer managed in a standardized care pathway was associated with an improvement in tumour characteristics

**DOI:** 10.1002/bco2.301

**Published:** 2023-10-06

**Authors:** Suleiman Abuhasanein, Staffan Jahnson, Henrik Kjölhede

**Affiliations:** ^1^ Department of Urology, Institute of Clinical Science, Sahlgrenska Academy University of Gothenburg Göteborg Sweden; ^2^ Department of Surgery, Urology Section NU Hospital Group Uddevalla Sweden; ^3^ Department of Clinical and Experimental Medicine, Division of Urology Linköping University Linköping Sweden; ^4^ Department of Urology Sahlgrenska University Hospital Göteborg Sweden

**Keywords:** bladder cancer, haematuria, resection of tumour in the urinary bladder, standardized care pathways, transurethral, trends

## Abstract

**Objectives:**

To evaluate whether the implementation of standardized care pathway (SCP) for patients with suspected urinary bladder cancer (UBC) was associated with changes in tumour characteristics. Additionally, the study aims to explore whether there was a shift in the selection of patients prioritized for immediate evaluation regarding suspicion of UBC.

**Materials and Methods:**

The study included all patients diagnosed with UBC in the NU Hospital Group between 2010 and 2019. To evaluate changes associated with SCP, patients were divided into two diagnostic time periods, either before (2010–2015) or during (2016–2019) the implementation of the SCP. To evaluate which patients were prioritized for prompt evaluation within 13 days, logistic regression analysis was performed on all patients before and during SCP.

**Results:**

Median time to transurethral resection of the tumour in urinary bladder (TURBT) decreased from 29 days (interquartile range [IQR] 16–48) before SCP to 12 days (IQR 8–19) during SCP (*p* < 0.001) with a clear break from 2016. The proportion of cT2 + tumours decreased during SCP from 26% to 20% (*p* = 0.035). In addition, tumours detected during SCP were smaller (*p* = 0.023), but with more multiple lesions (*p* = 0.055) and G3 tumours (*p* = 0.007). During SCP, there was no statistically significant difference between the groups of patients with TURBT within or after 13 days. In contrast, before SCP, a majority of the patients treated within 13 days had advanced tumours and were admitted from the emergency ward.

**Conclusions:**

The implementation of an SCP for suspected UBC was associated with improved tumour characteristics. Interestingly, during SCP, there were no substantial differences in patients' or tumours' characteristics among those who underwent TURBT within or after 13 days. This indicates that the 13‐day timeframe for TURBT might be prolonged, especially in less urgent cases in order to facilitate a prioritization of more severe cases with treatable disease.

## 1 INTRODUCTION

Urinary bladder cancer (UBC) is one of the most common malignancies.^1^ The incidence of UBC has shown variations over time worldwide.^2^ A delay in UBC diagnosis is associated with a worse outcome.^3^, ^4^, ^5^ However, a short time from symptom to referral has been associated with a better outcome, while a short time from referral to transurethral resection of tumour in the bladder (TURBT) has been associated with more advanced tumours and worse outcome, indicating that patients' delay and delay in primary care units may be more important for prognosis than the hospital delay.^6^

To improve early diagnosis of cancer, various strategies have been implemented, such as raising public awareness about the importance of seeking medical care for alarm symptoms and educating of primary care physicians to accurately assess patients and refer them for further evaluation following established guideline.^7^ In Sweden, a standardized care pathway (SCP) for patients with suspected UBC was implemented nationwide in 2016. The SCP included immediate referral from primary care units of patients with macroscopic haematuria, cystoscopy and computed tomography urography (CTU).^8^ In addition, the SCP was accompanied with an educational campaign for primary care physicians to increase awareness of macroscopic haematuria as a symptom to be promptly evaluated.

During the first 4 years after the implementation of SCP, the median time from referral to TURBT nationally was shortened from 37 to 27 days indicating a limited adherence to the SCP guidelines.^9^ The effect of the SCP could therefore not be evaluated on a national basis, as it was not applied similarly in a determined and consistent manner in all regions. Therefore, the objective of this study was to examine the impact of the implementation of SCP with shortened time to diagnosis for patients with suspected UBC in a healthcare institution where the SCP guidelines were widely followed. The aim was to investigate whether this implementation was associated with changes in tumour characteristics and whether there was a shift in the non‐scheduled selection of patients who were given priority for prompt evaluation.

## 2 MATERIALS AND METHODS

### 2.1 Patients

All patients with newly diagnosed UBC between 1st January 2010 and 31st December 2019 in NU Hospital Group (in the western region of Sweden serving a population of 290 000 patients) were included. Data were collected retrospectively from the medical records and analysed regarding patients' age and gender, tumour characteristics such as tumour size (registered as the largest diameter of the largest tumour), number of tumours, clinical stage according to TNM, 8th edition^10^ and tumour grade according to the WHO 1999 classification.^11^ If two grades were reported in the pathology report, the more severe grade was recorded. In addition, treatment variables were registered, such as primary treatment after TURBT in terms of second‐look resection (SLR—defined as re‐resection within 56 days after TURBT), intravesical instillation therapy (IVIT—defined as multiple intravesical instillations of BCG or Mitomycin C, excluding immediate postoperative single instillations), cystectomy and whether patients were discussed at a multidisciplinary team conference (MDTC). Final clinical TNM stage was registered after SLR and discussion in MDTC.

Furthermore, the patients were classified either as having been referred from a primary care unit for the SCP, or starting the SCP in conjunction with an emergency admission, often due to severe macroscopic haematuria with clotting. The reason for starting the SCP for each patient was classified as either macroscopic haematuria, cystoscopy for other reasons, or incidental findings on radiology for other reasons.

### 2.2 Standardized care pathway

SCP was implemented in 2016 at the NU Hospital Group by establishing an outpatient clinical practice where all patients who fulfilled the SCP criteria underwent CTU and cystoscopy on the same day. The criteria for SCP were suspicion of UBC on any diagnostic imaging done for other reasons regardless of age, or macroscopic (but not microscopic) haematuria in patients aged more than 50 years. A lower age limit of 40 years was initially used but was changed in 2018 due to the rarity of UBC between 40 and 50 years. All patients fulfilling the SCP criteria should be referred within 24 h from primary care units or from other care units and undergo CTU and cystoscopy within 7 days, although information about the time from first symptom to referral was not available in this study. From the referral date, TURBT should be performed for all eligible patients within no more than 13 days. Furthermore, all patients with cT1 + tumours should be discussed at MDTC within 26 days.

### 2.3 Statistics

To evaluate changes related to SCP, patients were divided into two diagnostic time periods, either before (2010–2015) or during (2016–2019) the implementation of the SCP. Furthermore, time trends unrelated to SCP were assessed by analysing four time periods: 2010–2012 and 2013–2015 (prior to the SCP implementation) and 2016–2017 and 2018–2019 (after the SCP implementation). Age and time periods were described using median and interquartile range (IQR). Differences between groups were compared using *χ* ^2^ test for categorical variables or the Wilcoxon signed‐rank test for continuous variables.

To evaluate which patients were prioritized for prompt evaluation, logistic regression analysis was performed on all patients into two multivariate analyses (before and during the implementation of SCP) using the time from the date of referral to the date of TURBT (time to TURBT), either ≤13 days or >13 days—according to the time limit indicated by the recommendations for SCP—as the dependent variable. Age, admission modality, tumour size, tumour multiplicity and stage were used as explanatory variables. *p* values <0.05 were considered statistically significant. Statistical analysis was performed using SPSS version 29 (IBM Corp., Armonk, NY, USA).

## 3 RESULTS

Between 1 January 2010 and 31 December 2019, 785 patients were diagnosed with UBC in the NU Hospital Group, of whom 455 (58%) were diagnosed before SCP and 330 (42%) during SCP (Table 1). The median age was 74 years (IQR 68–81) before SCP compared with 75 years (IQR 70–82) during SCP (*p* = 0.148). The proportion of referrals from primary care units was unchanged (82% to 83%, *p* = 0.575). There was an increase in the proportion of patients managed according to guidelines, with discussion at MDTC of cT1 + tumours (2% to 89%, *p* < 0.001), SLR in cT1 tumours (29% to 52%, *p* < 0.001), and IVIT of eligible patients (64% to 77%).

**TABLE 1:** Descriptive parameters of all patients diagnosed with urinary bladder cancer in the NU Hospital Group between 2010 and 2019 stratified into two time periods in relation to the implementation of SCP.

Variable name		Before SCP 2010–2015	During SCP 2016–2019	All	*p* value
No. patients	(% of the row)	455 (58)	330 (42)	785	
Gender, *n* (%)	Male	357 (79)	245 (74)	602 (77)	0.169
Age (years)	Median, (IQR)	74 (67–81)	75 (70–82)	75 (68–81)	0.148
Age, *n* (%)	≤75 years	241 (53)	167 (51)	308 (52)	0.531
Admission modality	Referral	371 (82)	273 (83)	644 (82)	0.575
	Emergency	69 (15)	43 (13)	112 (14)	
	Others	15 (3)	14 (4)	29 (4)	
Reason for investigation	Macroscopic haematuria	350 (77)	259 (78)	609 (78)	0.605
	Others^a^	105 (23)	71 (22)	176 (22)	
Number of tumours	Single	306 (67)	200 (61)	506 (64)	0.055
	Multiple	149 (33)	130 (39)	279 (36)	
Tumour size	≤30 mm	193 (59)	196 (67)	389 (63)	0.023^b^
	>30 mm	137 (41)	95 (33)	232 (37)	
	missing	125 (28)	39 (12)	164 (20)	
Tumour grade, *n* (%)	G1 + G2	315 (69)	198 (60)	513 (65)	0.007
	G3	140 (31)	132 (40)	272 (35)	
cT, *n* (%)^c^	TaG1‐2	192 (42)	163 (49)	355 (45)	0.046
	TaG3, Tis, T1	144 (32)	102 (31)	246 (31)	0.826
	T2+	119 (26)	65 (20)	184 (24)	0.035
cN, *n* (%)	N+	7 (2)	10 (3)	17 (2)	0.156
cM, *n* (%)	M1	7 (2)	5 (2)	12 (2)	0.979
Intra‐vesical instillation therapy, *n* (%)	For eligible patients	82 (64)	56 (77)	138 (69)	0.063
Second look resection, *n* (%)	For cT1	37 (29)	38 (52)	75 (37)	0.001
Multi‐disciplinary team conference, *n* (%)	For cT1+	4 (2)	132 (89)	136 (34)	<0.001
Cystectomy, *n* (%)	For cT2+	61 (51)	31 (48)	92 (50)	0.644
Time to TURBT (days)	Median, (IQR)	29 (16–48)	12 (8–19)	21 (10–38)	<0.001
Time to TURBT, *n* (%)	0–13 days	94 (21)	187 (57)	281 (36)	<0.001
	>13 days	361 (79)	143 (43)	504 (64)	

*Note*: Figures represent number of patients (% of the column) if not otherwise indicated.

Abbreviations: IQR, interquartile range; SCP, standardized care pathway; TURBT, resection of tumour in urinary bladder; UBC, urinary bladder cancer.

^a^ This includes bladder cancer detected because of other reasons than macroscopic haematuria.

^b^ Missing values were excluded from Chi2 analysis.

^c^ TaG1‐2 tested against all other T categories together; TaG3, TIS and T1 tested against all other T categories together; T2+ tested against all other T categories together.

Time trends showed no increase in discussion at MDTC before SCP, while the use of IVIT and SLR was increasing already before the introduction of SCP (Table S1). There was no change in the proportion of patients with cT2 + tumours treated with cystectomy before and during SCP (51% compared with 48% respectively, *p* = 0.644), but there was a wide variation in these two periods.

**FIGURE 1:**
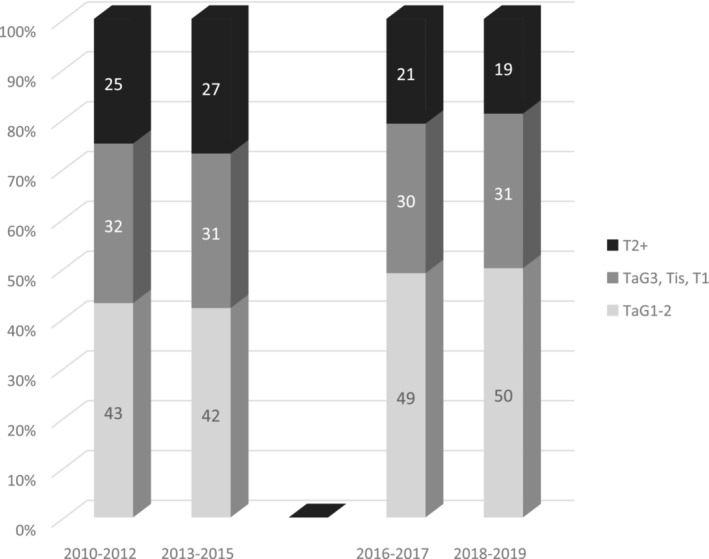
Time trends of tumour stages in all patients diagnosed with urinary bladder cancer in NU Hospital Group between 2010 and 2019 stratified into four time periods.

Median time to TURBT decreased from 29 days (IQR 16–48) to 12 days (IQR 8–19) (*p* < 0.001) with a clear change in 2016. The proportion of patients who had TURBT within 13 days increased from 21% before SCP to 57% (*p* < 0.001) during SCP. The proportion of cTaG1‐2 also changed over time, from 42% before SCP to 49% during SCP (*p* = 0.046), with a corresponding decrease in the proportion of cT2 + tumours (26% to 20% respectively, *p* = 0.035), while time trends showed a clear‐cut change with the introduction of SCP (Figure 1). In addition, tumours detected during SCP were smaller (*p* = 0.023), although with a larger proportion of missing data before SCP, but with more multiple lesions (*p* = 0.055) and G3 tumours (*p* = 0.007).

**FIGURE 2:**
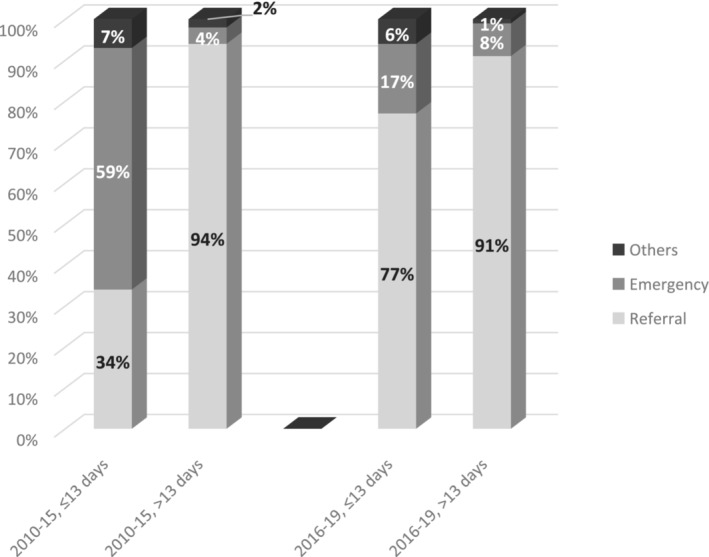
Time trends of admission modality in all patients with urinary bladder cancer in NU Hospital Group between 2010 and 2019 stratified into four groups regarding to time from referral to transurethral resection of tumour in urinary bladder (with cut‐off value of 13 days) and the relation to the implementation of the standardized care pathway.

Within 13 days and before the introduction of SCP, the majority (59%) of patients had an emergency admission, while this proportion decreased to 17% during SCP, and the proportion of patients managed after referral from other care units increased from 34% to 77% (*p* < 0.001) (Table 2 and Figure 2). Within 13 days and before SCP, patients with cT2 + tumours had a lower rate of treatment with cystectomy 32%, compared with 56% during SCP (*p* = 0.018), suggesting that most of the patients before SCP had more advanced tumours (Table 2). Similarly, patients with TURBT within 13 days before compared with during SCP, had more often large tumours >3 cm (57% vs. 35%, *p* = 0.003). The proportion of cT2 + tumours evaluated within 13 days decreased from 43% before SCP to 21% during SCP (*p* < 0.001). Similarly, within 13 days, before compared with during of SCP, the proportion of patients with cTaG1‐2 increased from 35% to 46%.

**TABLE 2:** Descriptive parameters of all patients with urinary bladder cancer in NU Hospital Group between 2010 and 2019 stratified into four groups regarding to time to TURBT and the relation to the implementation of SCP.

Variable name		Before SCP 2010–2015			During SCP 2016–2019		
		0–13 days	>13 days	*p* value	0–13 days	>13 days	*p* value
No. patients	(% of the row)	94 (21)	361 (79)		187 (57)	143 (43)	
Gender, *n* (%)	Male	73 (78)	284 (79)	0.832	144 (77)	101 (71)	0.189
Age (years)	Median, (IQR)	77 (67–86)	75 (67–81)	0.083	75 (69–81)	77 (71–83)	0.023
Age, *n* (%)	≤75 years	41 (46)	181 (52)	0.253	95 (53)	59 (43)	0.108
Admission modality	Referral	32 (34)	339 (94)	<0.001	143 (77)	130 (91)	0.002
	Emergency	55 (59)	14 (4)		32 (17)	11 (8)	
	Others	7 (7)	8 (2)		12 (6)	2 (1)	
Reason for investigation	Macroscopic haematuria	65 (69)	285 (79)	0.045	150 (80)	109 (76)	0.382
	Others^a^	29 (31)	76 (21)		37 (20)	34 (24)	
Number of tumours	Single	70 (74)	236 (65)	0.094	116 (62)	84 (59)	0.544
	Multiple	24 (26)	125 (35)		71 (38)	59 (41)	
Tumour size	≤30 mm	27 (43)	166 (62)	0.005	107 (65)	89 (71)	0.297
	>30 mm	36 (57)	101 (38)		58 (35)	37 (29)	
	Missing	31 (33)	94 (26)		22 (12)	17 (12)	
Tumour grade, *n* (%)	G1 + G2	58 (62)	257 (71)	0.076	107 (57)	91 (64)	0.238
	G3	36 (38)	104 (29)		80 (43)	52 (36)	
cT, *n* (%)	TaG1–2	33 (35)	159 (44)	<0.001	87 (46)	76 (53)	0.491
	TaG3, Tis, T1	21 (22)	123 (34)		61 (33)	41 (29)	
	T2+	40 (43)	79 (22)		39 (21)	26 (18)	
cN, *n* (%)	N+	2 (2)	5 (2)	0.602	5 (3)	5 (3)	0.666
cM, *n* (%)	M1	2 (2)	5 (2)	0.602	3 (2)	2 (2)	0.880
Intra‐vesical instillation therapy, *n* (%)	For eligible patients	10 (48)	72 (67)	0.086	33 (79)	23 (74)	0.662
Second look resection, *n* (%)	For cT1	7 (33)	30 (28)	0.637	25 (60)	13 (42)	0.137
Multi‐disciplinary team conference, *n* (%)	For cT1+	3 (5)	1 (1)	0.019	73 (84)	59 (95)	0.033
Cystectomy, *n* (%)	For cT2+	13 (32)	51 (62)	0.001	22 (56)	9 (35)	0.085

*Note*: Figures represent number of patients (% of the column) if not otherwise indicated.

Abbreviations: IQR, interquartile range; SCP, standardized care pathway; TURBT, resection of tumour in urinary bladder.

^a^ This includes bladder cancer detected because of incidental findings on medical imaging or other symptoms than macroscopic haematuria.

Logistic regression analysis of the patients diagnosed during SCP showed no statistically significant explanatory variables between groups having TURBT within or after 13 days regarding age, tumour size, multiplicity, admission modality and stage (Table 3). However, before SCP, emergency admission was statistically significant independent predictor (*p* < 0.001) for patients having TURBT within 13 days.

**TABLE 3:** Two multivariate logistic regression analyses performed on all patients with urinary bladder cancer in NU Hospital Group (before and after the implementation of standardized care pathway) using the dichotomized time to transurethral resection of the tumour in urinary bladder (≤13 days or >13 days) as the dependent variable.

Variable name	Before SCP 2010–2015		During SCP 2016–2019	
	HR multivariate (95 %CI)	*p* value	HR multivariate (95 %CI)	*p* value
Age groups	1		1	
≥75 years	1.81 (0.90–3.63)	0.097	1.49 (0.92–2.40)	0.103
Number of tumours, single	1		1	
Multiple	0.85 (0.41–1.70)	0.621	1.21 (0.75–1.97)	0.439
Tumour size	1		1	
>30 mm	0.75 (0.34–1.63)	0.464	0.88 (0.47–1.45)	0.507
Admission modality	1		1	
Emergency	0.04 (0.02–0.10)	<0.001	0.52 (0.23–1.14)	0.102
TNM	cTaG1–2	1		1
cTaG3, Tis, T1	1.64 (0.70–3.86)	0.254	0.74 (0.43–1.30)	0.291
cT2+	1.01 (0.40–2.52)	0.985	1.11 (0.54–2.27)	0.744

Abbreviations: CI, confidence interval; HR, hazard ratio; SCP, standardized care pathway; TNM, tumour, lymph node, metastasis.

## 4 DISCUSSION

In this study, we assessed a recently implemented SCP for individuals with suspected UBC at an institution where the SCP guidelines were more widely followed than in the recent national evaluation.^9^ We observed a decrease in median time from referral to TURBT from 29 to 12 days, a decrease in the proportion of cT2 + tumours from 26% to 20%, a corresponding increase in cTaG1‐2 from 42% to 49%, and a decrease in the proportion of larger tumours >3 cm from 41% to 33%. Also, during SCP, no significant variations were detected in patients' or tumours' characteristics between those who underwent TURBT within or after 13 days whereas before SCP emergency admission was the main driver for fast evaluation.

The decrease in time to TURBT was more pronounced in this study than in the national study we previously reported on, where time from referral to TURBT decreased from 37 to 27 days.^9^ However, despite the streamlined process and dedicated outpatient clinic, half of the patients did not undergo TURBT within the recommended 13‐day timeframe, suggesting that meeting this target was challenging and may not be feasible. Also, while the guidelines were mostly followed only 52% of patients with cT1 tumours underwent SLR, leaving room for improvements.

The observed improved clinical stage during SCP in this study may not only be due to management after referral, but the time before referral might also be important. The attention around the introduction of the SCP may also possibly have shortened the time from first symptom to referral, although this could not be studied in this study. In a large surveillance, epidemiology and end results (SEER) study by Hollenbeck et al.^12^ found that a delay between the onset of the first symptom and diagnosis of UBC was linked to a greater risk of cancer‐specific death, underscoring the importance of promptly referring patients for specialized urological evaluation. Similarly, Wallace et al.^6^ observed that a delay between symptom and referral was more important for prognosis than time from referral to TURBT. However, in the present study and after the implementation of SCP, patients should be referred within 24 h from primary care making the former delay probably short.

There are several factors that can contribute to delays in management of UBC including lack of awareness or knowledge from the public about disease symptoms, and delay in diagnostic testing and referral to specialists.^12^, ^13^, ^14^, ^15^ Socioeconomic and demographic factors, such as low‐income and minority status, may also play a role in delay to treatment.^15^ Therefore, SCP may be important to raise public awareness about the disease, improve education in the primary care units and implement systems to streamline the diagnostic and referral process for patients with suspected UBC.^7^, ^9^ On the other hand, a shorter time from referral to TURBT does not necessarily result in a shorter time to definitive treatment with, for example, cystectomy,^16^, ^17^ which emphasizes the importance of taking the entire process from symptom to definitive treatment into account.

During SCP, a higher proportion of cT2 + patients had cystectomy in the group with TURBT within 13 days compared with after 13 days (56% vs. 32%). Similar trends were noticed for the cT1 group treated with SLR and IVIT. These observations seem to indicate some degree of prioritization of patients for further treatment after SCP. In the current study, a higher proportion of G3 tumours was observed during SCP, which was similar to the national SCP series.^9^ These results seem to contradict the primary findings; however, this could be attributed to a shift in the assessment of tumour grade at the local pathology department, possibly influenced by a general trend towards assigning higher grades (G3) to UBC.^18^

We did not notice any difference in tumour aggressiveness between the groups having TURBT within or after 13 days. Whereas emergency admission was the only identified statistically significant independent predictor for patients having TURBT within 13 days, no difference was found in logistic regression analysis comparing age, tumour size, number and stage in patients both before and during SCP. Before SCP implementation, more than half of the patients who were treated within 13 days were emergency cases. These patients may have had more advanced tumours, which could explain the higher percentage of larger and cT2 + tumours in the group treated within 13 days, as compared with those treated after 13 days. This observation is supported by the fact that only 32% of patients with cT2 + before SCP and within 13 days underwent cystectomy, compared with 56% during SCP. However, similar differences regarding emergency admission were not observed during SCP. Based on these observations, it appears that, rather than imposing a brief time constraint for all patients undergoing TURBT, prioritizing timely definitive treatment may be particularly crucial for patients with advanced tumours beyond TaG1‐2, which necessitates a meticulous selection of this cohort for more intricate management.^6^, ^17^

The main strength of this study is that the SCP was mostly conducted as intended, with a majority of the patients undergoing TURBT within the recommended time limit and a noticed improvement in management according to guidelines. Moreover, this is one of the largest reported series of patients undergoing investigation for suspected UBC with an SCP. Nevertheless, there are several shortcomings in our study. Because the data were collected retrospectively, there may be several potential biases. However, analysing the periods before and during SCP may suggest a correlation with changes in tumour characteristics. Other potential limitations of our study are lacking data about time from symptoms to referral, short observation time and lack of information on cancer‐specific survival. Further follow‐up should improve the understanding of the effects of the SCP.

## 5 CONCLUSIONS

The implementation of an SCP for suspected UBC was associated with improved tumour characteristics suggesting that a well implemented SCP and general awareness of alarm symptoms of UBC might in the future improve outcomes for UBC patients. Notably, during SCP, no significant variations were detected in patients' or tumours' characteristics between those who underwent TURBT within or after 13 days, suggesting that the 13‐day timeframe for TURBT could potentially be extended, especially in less urgent cases in order to facilitate a prioritization of more severe cases with treatable disease.

## AUTHOR CONTRIBUTIONS

**Suleiman Abuhasanein**: Conceptualization; methodology; formal analysis; data curation; writing the original draft preparation. **Staffan Jahnson**: Supervision; methodology; data curation; writing the original draft preparation. **Henrik Kjölhede**: Supervision; data curation; writing review and editing.

## CONFLICT OF INTEREST STATEMENT

The authors have no conflict of interest to declare.

## Supporting information


**Table S1.** Descriptive parameters in all patients with bladder cancer stratified into four time periods in relation to the implementation of the standardized care pathway. Figures represent number of patients (% of numbers of the column if not otherwise indicated). (IQR: interquartile range, TURBT: transurethral resection of tumour in urinary bladder).
