# Supplementary material for: Shortened time to diagnosis for patients suspected of urinary bladder cancer managed in a standardized care pathway was associated with an improvement in tumour characteristics
Source: BJUI Compass. 2023 Oct 6;5(2):261–8. doi: 10.1002/bco2.301 (PMC10869653; doi:10.1002/bco2.301)
Supplement: Supplementary file 1 — Table S1. Descriptive parameters in all patients with bladder cancer stratified into four time periods in relation to the implementation of the standardized care pathway. Figures represent number of patients (% of numbers of the column if not otherwise indicated). (IQR: interquartile range, TURBT: transurethral resection of tumour in urinary bladder). [file BCO2-5-261-s001.docx]

**Supplementary Table 1. Descriptive parameters in all patients with bladder cancer stratified into four time periods in relation to the implementation of the standardized care pathway. Figures represent number of patients (% of numbers of the column if not otherwise indicated).** (IQR: interquartile range, TURBT: transurethral resection of tumor in urinary bladder)

| **Variable name** | | **Before SCP** | | **During SCP** | |
| --- | --- | --- | --- | --- | --- |
|  |  | **2010-2012** | **2013-2015** | **2016-2017** | **2018-2019** |
| **No. patients** | **(% of the row)** | 179 (23) | 276 (35) | 164 (21) | 166 (21) |
| **Gender, n (%)** | **Male** | 140 (78) | 217 (79) | 121 (74) | 124 (75) |
| **Age (years)** | **Median, (IQR)** | 75 (66-81) | 76 (68-81) | 75 (69-82) | 77 (70-82) |
| **Age, n (%)** | **≤ 75 yrs.** | 97 (54) | 144(52) | 89 (54) | 78 (47) |
| **Admission modality** | **Referral** | 143 (80) | 228 (83) | 143 (87) | 130 (78) |
|  | **Emergent** | 29 (16) | 40 (14) | 13 (8) | 30 (18) |
|  | **Others** | 7 (4) | 8 (3) | 8 (5) | 6 (4) |
| **Suspected UBC** | **Macroscopic hematuria** | 136 (76) | 214 (77) | 130 (80) | 129 (78) |
|  | **Others*** | 43 (24) | 62 (23) | 34 (21) | 37 (22) |
| **Number of tumors** | **Single** | 131 (73) | 175 (63) | 101 (62) | 99 (60) |
|  | **Multiple** | 48 (27) | 101 (37) | 63 (38) | 67 (40) |
| **Tumor size** | **≤30 mm** | 60 (52) | 133 (62) | 100 (70) | 96 (65) |
|  | **>30 mm** | 56 (48) | 81 (38) | 43 (30) | 52 (35) |
|  | **Missing** | 63 (35) | 62 (23) | 21 (13) | 18 (11) |
| **Tumor grade, n (%)** | **G1+G2** | 126 (70) | 189 (69) | 100 (61) | 98 (59) |
|  | **G3** | 53 (30) | 87 (31) | 64 (39) | 68 (41) |
| **cT, n (%)** | **TaG1-2** | 77 (43) | 115 (42) | 80 (49) | 83 (50) |
|  | **TaG3, Tis, T1** | 58 (32) | 86 (31) | 50 (30) | 52 (31) |
|  | **T2+** | 44 (25) | 75 (27) | 34 (21) | 31 (19) |
| **cN, n (%)** | **N+** | 1 (1) | 6 (2) | 7 (4) | 3 (2) |
| **cM, n (%)** | **M1** | 1 (1) | 6 (2) | 2 (1) | 3 (2) |
| **Intra-vesical instillation therapy, n (%)** | **For eligible patients** | 32 (60) | 50 (67) | 27 (79) | 29 (74) |
| **Second look resection, n (%)** | **For cT1** | 13 (25) | 24 (32) | 20 (59) | 18 (46) |
| **Multi-disciplinary team conference, n (%)** | **For cT1+** | 0 (0) | 4 (3) | 63 (86) | 69 (91) |
| **Cystectomy, n (%)** | **For cT2+** | 18 (41) | 43 (57) | 19 (56) | 12 (39) |
| **Time to TURBT (days)** | **Median, (IQR)** | 34 (21-55) | 28 (14-44) | 11 (7-20) | 13 (9-19) |
| **Time to TURBT, n (%)** | **0-13 days** | 29 (16) | 65 (24) | 97 (59) | 90 (54) |
|  | **>13 days** | 150 (84) | 211 (76) | 67 (41) | 76 (46) |

*This includes bladder cancer detected because of incidental findings on medical imaging or for other symptoms than macroscopic hematuria.
